# The impact of life events on adult physical and mental health and well-being: longitudinal analysis using the GoWell health and well-being survey

**DOI:** 10.1186/s13104-016-2278-x

**Published:** 2016-10-18

**Authors:** Claire Cleland, Ade Kearns, Carol Tannahill, Anne Ellaway

**Affiliations:** 1 School of Natural and Built Environment, David Keir Building, Stranmillis Road, Belfast, UK; 2 Urban Studies, School of Social and Political Sciences, University of Glasgow, Glasgow, UK; 3 Glasgow Centre for Population Health, Glasgow, UK; 4 MRC/CSO Social and Public Health Sciences Unit, University of Glasgow, 200 Renfield Street, Glasgow, UK

**Keywords:** Life events, Physical health, Mental health, Mental well-being, GoWell, Longitudinal analysis

## Abstract

**Background:**

It is recognised that life events (LEs) which have been defined as incidents necessitating adjustment to habitual life either permanently or temporarily, not only have the potential to be detrimental to health and well-being, but research suggests some LEs may be beneficial. This study aimed to determine the individual and cumulative occurrence of LEs; and to establish their effect on health and well-being.

**Results:**

Demographic factors (gender, age and highest educational attainment), LE occurrence and self-reported health data were collected as part of the longitudinal GoWell community health and wellbeing survey (2008–2011). Self-reported health was measured using the SF-12 questionnaire for physical (SF-12 PCS) and mental health (SF-12 MCS) and the Warwick–Edinburgh mental well-being scale (WEMWBS) for well-being. Statistical analysis was performed using SPSSv21 and level of significance was set at *p* < 0.05. Results showed that the sample was 61.6 % (n = 768) female; 20.4 % (n = 254) were aged 16–39 years, 46.1 % (n = 575) 40–64 years and 33.5 % (n = 418) were over 65 years; 68.8 % (n = 819) had no qualifications/Scottish leaving certificates, with the remaining 31.2 % (n = 372) having their highest educational qualification above Scottish leaving certificates. Health score means were 49.3 SF-12 mental health component score (SF-12 MCS); 42.1 SF-12 physical health component score (SF-12 PCS); and 49.2 WEMWBS. Participants experienced 0–7 LEs over a three year period, with the most common being: housing improvement (44.9 %), house move (36.8 %), health event (26.3 %) and bereavement (25.0 %). Overall, an increase in LEs was associated with a health score decrease. Five LEs (relationship breakdown, health event, bereavement, victimisation and house move) had negative impacts on SF-12 MCS and two (new job/promotion and parenthood) had positive impacts. For SF-12 PCS only three (health event, bereavement and housing improvement) had a negative impact. Six (health event, victimisation, bereavement, relationship breakdown housing move and improvement) had negative impacts on well-being and two (new job/promotion, marriage) had positive effects.

**Conclusions:**

Findings from the current study confirm LEs have both detrimental and beneficial impacts on health and well-being. Further research is required to disentangle the complexity of LEs and the ways they affect health and well-being.

## Background

In recent decades, research into life events (LEs) and health has burgeoned, and while early work assumed all LEs were potentially health-damaging, it is increasingly recognised this may be inaccurate, and some LEs may be potentially beneficial [1–7]. LEs are defined as incidents that can significantly interfere with ongoing life, necessitating adjustment to habitual life either temporarily or on a permanent basis [1, 7–10]. For example: death of a close relative or friend; marriage; birth of a child; redundancy; new home; or a new job [7, 8, 11].

Published literature reviewing the aetiology of disease has indicated that LEs can play a role in the development of non-communicable diseases as the physiological and biological processes that individuals may experience due to a LE, may heighten their susceptibility to develop acute and/or chronic life threatening conditions as a consequence of stress and its mediating factors [1]. In addition to the direct impacts that LEs may have on the individual and a potential susceptibility to developing non-communicable diseases, recent research has provided evidence of the indirect detrimental impacts that LEs can have on the health and well-being of the individual via the uptake of unhealthy lifestyle behaviours, particularly poor and inadequate dietary habits, increased alcohol intake and/or smoking [4–7]. The occurrence of LEs has also been found to impact well-being [4]. Well-being not only refers to the ability of an individual to realise their potential, cope with normal life stresses and to work productively, making a contribution to society; but within the organisation for economic co-operation and development well-being indicators, ‘Quality of life’ (health status, work and life balance, education and skills, social connections, environmental quality, civic engagement, personal security and subjective well-being) and ‘Material Living Condition’ (income and wealth, jobs and earnings and housing) are also considered [12, 13].

Previous reports have found that people classified as ‘disadvantaged’ experienced a greater number of LEs in comparison to those more affluent, this being the case specifically for crime, unemployment, redundancy and bereavement, contributing to widening health inequalities [14–16]. However, few studies have explored the health effects of cumulative exposure to LEs; and the magnitude and direction of effect in relation to health and well-being for so-called ‘desirable’ LEs (marriage, new job/promotion) and for those LEs which are challenging to classify (retirement, parenthood, divorce, relationship breakdown) and which may cause a range of contradictory emotions [7, 8, 17, 18].

The aim of this study was threefold: to determine the occurrence of LEs in an adult population in the United Kingdom; to establish the cumulative occurrence of LEs and its effect on health and well-being; and to investigate the extent and direction of effect that each LE has on health and well-being.

## Methods

### Study population

GoWell is a large collaborative regeneration study that commenced in 2006 and is being implemented across Glasgow for a period of 10 years [19]. Glasgow is a suitable location for this regeneration project on account of its high levels of socio-economic disadvantage, considerable health inequalities and low levels of life expectancy [11, 20–22]. The overarching aim of the project is to investigate the impact of area and housing regeneration on the health and well-being of residents and their communities in order to inform research, policy and practice [19].

### Data source

Data were collected at three time points by the repeat cross-sectional GoWell community health and wellbeing survey (wave 1, 2006; wave 2, 2008; and wave 3, 2011). Eligibility criteria included: (1) at least 16 years of age; (2) currently paying a mortgage, owned their own home, a social sector tenant or private sector leaseholder; and (3) were either the sole or main adult resident residing in the household, or that person’s partner.

The data analysed in the current study were from the GoWell nested longitudinal cohort (wave 2, 2008 and wave 3, 2011; n = 1247) [19]. Participants completed written informed consent prior to their face-to-face interview which included questions regarding: demographics; the condition of their home; their neighbourhood perceptions; amenity use; health behaviours; and their health and well-being.

### Demographic characteristics

Within the current study three demographic categorical characteristics recorded at wave 3 were of interest: gender (male or female); age group (16–24; 25–39; 40–54; 55–64; or 65+ years); and highest educational attainment (none/Scottish Leaving Certificates; or higher than Scottish leaving certificates).

### Life events

Nine LEs were included in the Wave 3 survey following a review of the literature [3–5, 7]. Participants were asked (yes/no) if they had been affected by any of the LEs in the previous three years: (1) serious health event, illness or disability affecting you or another household member; (2) new job/promotion; (3) unemployment, redundancy or reduced working hours; (4) you or your partner became pregnant or you became a parent; (5) serious problem with or break-up of relationship with partner; (6) death of someone close; (7) marriage or setting up home with a partner; (8) being the victim of a crime; (9) behavioural problem with a child at home or problem at school; where someone did not have children, this life event was recorded as negative, i.e. not experienced. In addition, respondents were asked separately whether they had improvements carried out to their home in the past 3 years, and whether they lived at the same or a different address three years previously. From these responses, we included two further housing-related life events: (10) receipt of housing improvements; and (11) moved house.

### Health outcome measures

In order to assess health over time and to investigate the extent of change measures of physical health, mental health and mental well-being were incorporated at both time-points (wave 2 and 3).

#### Physical and mental health outcomes

The SF-12v2 Health Survey was used as an alternative to the lengthier SF-36. The SF-12 has been found to have a high degree of correspondence to the SF-36 with product-moment correlations ranging from 0.94 to 0.96 and 0.94 to 0.97 for the physical and mental measures consecutively [18, 23, 24].

The SF-12 consists of twelve items that are formulated on eight subscales. The scores collected for each subscale were combined to provide two overall scores: physical component score (PCS) and mental component score (MCS). The PCS is made up of scores relating to physical functioning, bodily pain, general health and role limitations due to physical health [23, 24]. An example of questions include: “During the past 4 weeks, have you had any of the following problems with your work or other regular daily activities as a result of your physical health?” “Accomplished less than you would like (yes/no)?” “Were limited in the kind of work or other activities (yes/no)?” The MCS is comprised of scores relating to vitality, mental health, social functioning and role limitations due to emotional health [23, 24]. Questions include: “During the past 4 weeks, have you had any of the following problems with your work or other regular daily activities as a result of any emotional problems (such as feeling depressed or anxious)?” “Accomplished less than you would like (yes/no)?” “Did work or activities less carefully than usual (yes/no)?” PCS and MCS scores can range from 0 to 100; a higher score equates to a better level of physical and/or mental health [25].

#### Mental well-being outcome

Mental well-being was measured by the Warwick–Edinburgh mental well-being scale (WEMWBS) [25]. This instrument assesses characteristics of mental well-being over the previous two weeks via the completion of 14 items on a five point Likert scale (none of the time, rarely, some of the time, often, all of the time) relating to positive outlooks [26, 27]. Questions that comprise the WEMWBS are scored positively and can range from 14 to 70. A higher score equates to more positive mental well-being [26]. Questions include: “I’ve been feeling optimistic about the future”; “I’ve been feeling useful”; and “I’ve had energy to spare”. Studies have shown that WEMWBS has good content validity, (Cronbach’s alpha score of 0.91, and test–retest reliability was high at 0.83) [26].

### Data analysis

Data analysis was performed using SPSS version 21. The first stage was to “clean” the data by removing participants who did not provide a dichotomous yes/no answer to the LE questions. Those ‘missing’ for the LE relating to having a child with behavioural problems were added to ‘no’ as it was assumed they did not have a child or did not have a child of an age that the question applied to.

The next stage of analysis involved: determining the patterning of LEs by demographic characteristics (gender, age and highest level of educational attainment); examining the cumulative occurrence of LEs; and then performing Kruskal–Wallis H non-parametric tests to establish any significant differences for SF-12 PCS, SF-12 MCS and WEMWBS between the cumulative levels of LE occurrence. Post-hoc analysis was also performed where a significant difference for the grouping variable was found. No controls were set in the analysis.

Subsequently, multivariate linear regressions were undertaken for each dependent variable to determine the proportion of variance explained by each grouping of independent variables. The same three stage analysis was followed for each dependent variable. Within the first block, the three independent demographic variables were entered; followed in the second block by the three baseline measures of health (recorded in 2008); and within the third block the eleven LEs were included.

Finally, a series of general linear models (GLMs) were formulated to establish whether there were significant differences in each of the three outcome scores between those who had, and those who had not, experienced each of the eleven LEs, examined separately. Gender, age, highest educational attainment and the baseline measures (recorded in 2008) of health were included in the models as controls for the effects of each LE. Each of the three baseline health measures were transformed from continuous to categorical standard deviation (SD) based tertiles to enable inclusion in both the analysis of variance and the GLMs: low (<−0.5 SD); moderate (−0.5 to +0.5 SD); and high (>+0.5 SD). Level of significance was set at *p* < 0.05.

## Results

The sample was 38.4 % male (n = 479) and 61.6 % female (n = 768); 20.4 % aged 16–39 years (n = 254), 46.1 % aged 40–64 years (n = 575) and 33.5 % were 65 years or over (n = 418); 68.8 % (n = 819) had no qualifications/Scottish leaving certificates as their highest educational attainment, with the remaining 31.2 % (n = 372) having their highest educational attainment above Scottish leaving certificates (Table 1).

**Table 1:** Participant demographic characteristics and outcome health scores

Demographic characteristic^a^	n (%)	Outcome measures^a^		
		SF-12 MCS	SF-12 PCS	WEMWBS
		Mean (SD)	Mean (SD)	Mean (SD)
*Gender*				
Male	479 (38.4)	50.6 (11.7)	41.4 (14.2)	49.4 (10.9)
Female	768 (61.6)	48.5 (11.9)	42.6 (14.9)	49.1 (10.2)
*Age*				
16–24	20 (1.6)	54.7 (9.0)	50.9 (11.0)	57.4 (7.8)
25–39	234 (18.8)	47.9 (12.5)	50.6 (11.1)	50.7 (10.6)
40–54	369 (29.6)	46.0 (12.9)	44.6 (13.2)	48.3 (11.9)
55–64	206 (16.5)	48.9 (11.6)	38.8 (14.1)	47.6 (9.6)
65+	418 (33.5)	53.0 (9.5)	36.1 (14.9)	49.6 (9.2)
*Education*				
None/SLC	819 (68.8)	49.4 (12.0)	39.7 (14.6)	48.3 (10.2)
>SLC	372 (31.2)	49.5 (11.5)	47.5 (13.3)	51.5 (10.8)
All	(1247)	49.3 (11.9)	42.1 (14.6)	49.2 (10.5)

*SD* standard deviation

^a^Data collected at wave 3, 2011

For each of the scores of the health outcome measure, health was poorer in this sample compared to those reported in the Scottish health survey (SF-12, 2003; WEMWBS, 2013) [28, 29]. For the SF-12 MCS, the current study found the mean value to be 49.3 (SD 11.9) and 42.1 (SD 14.6) for the SF-12 PCS in comparison to 52.0 and 49.0 for the Scottish health survey [29]. For WEMWBS results from the current study showed a mean score of 49.2 (SD 10.5) in comparison to a Scottish population mean of 50.0 [28].

On average males had higher SF-12 MCS and WEMWBS scores than females, whereas females had a higher SF-12 PCS. Results for age categories showed that with increasing age each health score decreased; although scores increased again for those aged >55 years for SF-12 MCS and 65+ for WEMWBS. In addition, scores for each of the three health outcome measures were higher among those who had a higher level of educational attainment (Table 1).

### LE occurrence

The most common LEs (experienced by more than 25 % of the sample over the past three years) were a housing improvement (44.9 %), housing move (36.8 %), serious health event, illness or disability affecting you or another household member (26.3 %), and the death of someone close (25.0 %). The least frequent LEs were marriage (1.4 %) and behavioural problem with a child at home or problem at school (2.5 %) (Table 2). Males were more likely to report a health event, new job/promotion, unemployment/redundancy/reduced hours, housing improvement and house move, whereas females more commonly reported parenthood, relationship breakdown, bereavement, marriage, victimisation and behavioural problem with a child. Health events were more common among older respondents. Younger people were more likely to experience a new job/promotion; unemployment/redundancy/reduced hours; marriage; and victimisation (Table 2). The occurrence of parenthood peaked within the 25-39 age categories. Those who had a higher level of education experienced more LEs of all sorts except health events and relationship breakdowns (Table 2).

**Table 2:** Life event occurrence by demographic characteristics

Demographic characteristics^a^	Life events^a^										
	Health event	New job or promotion	Unemployed, redundancy or reduced hours	Parenthood	Relationship breakdown	Bereavement	Marriage	Victimisation	Behavioural problem with child	Housing improvement	House move
*n (% of whole sample)*											
Total number	328 (26.3)	88 (7.1)	93 (7.5)	66 (5.3)	52 (4.2)	312 (25.0)	17 (1.4)	88 (7.1)	31 (2.5)	560 (44.9)	459 (36.8)
*n (% of those who experienced a LE)*											
Gender											
Male	133 (40.5)	38 (43.2)	50 (53.8)	20 (30.3)	17 (32.7)	116 (37.2)	5 (29.4)	31 (35.2)	3 (9.7)	231 (41.3)	190 (41.4)
Female	195 (59.5)	50 (56.8)	43 (46.2)	46 (69.7)	35 (67.3)	196 (62.8)	12 (70.6)	57 (64.8)	28 (90.3)	329 (58.8)	269 (58.6)
*Age*											
16–24	2 (0.6)	4 (4.5)	5 (5.4)	4 (6.1)	2 (3.8)	4 (1.3)	1 (5.9)	3 (3.4)	0 (0.0)	8 (1.4)	7 (1.5)
25–39	36 (11.0)	42 (47.7)	30 (32.3)	52 (78.8)	10 (19.2)	58 (18.6)	8 (47.1)	23 (26.1)	16 (51.6)	104 (18.6)	82 (17.9)
40–54	109 (33.2)	35 (39.8)	42 (45.2)	8 (12.1)	34 (65.4)	105 (33.7)	3 (17.6)	45 (51.1)	14 (45.2)	162 (28.9)	132 (28.8)
55–64	58 (17.7)	5 (5.7)	13 (14.0)	2 (3.0)	3 (5.8)	45 (14.4)	3 (17.6)	9 (10.2)	0 (0.0)	111 (19.8)	73 (15.9)
*65*+	123 (37.5)	2 (2.3)	3 (3.2)	0 (0.0)	3 (5.8)	100 (32.1)	2 (11.8)	8 (9.1)	1 (3.2)	175 (31.3)	165 (35.9)
*Education*											
None/SLC	231 (70.4)	25 (28.4)	40 (43.0)	30 (45.5)	34 (65.4)	184 (59.0)	5 (29.4)	41 (46.6)	13 (41.9)	351 (62.7)	290 (63.2)
>SLC	84 (25.6)	59 (67.0)	49 (52.7)	34 (51.5)	15 (28.8)	120 (38.5)	10 (58.8)	45 (51.1)	18 (58.1)	180 (32.1)	146 (31.8)
Missing	13 (4.0)	4 (4.5)	4 (4.3)	2 (3.0)	3 (5.8)	8 (2.6)	2 (11.8)	2 (2.3)	0 (0.0)	29 (5.2)	23 (5.0)

^a^Data collected at wave 3, 2011 for life events that had occurred since wave 2, 2008

### Cumulative occurrence of LEs

Individuals in the current study ranged from having none (n = 220, 20 %) to seven LEs (n = 1, 0.1 %) within the study period. The most common number of LEs was 1 (n = 343, 31.2 %) and >50 % of the sample had 1–2 LEs (n = 610, 55.5 %) (Table 3).

**Table 3:** Cumulative occurrence of LEs

Cumulative number of LE/s^a^	n	Percentage of sample	Cumulative percentage of sample
0	220	20.0	20.0
1	343	31.2	51.2
2	267	24.3	75.5
3	166	15.1	90.6
4	79	7.2	97.8
5	18	1.6	99.5
6	5	0.5	99.9
7	1	0.1	100.0

^a^Data collected at wave 3, 2011

Overall, a general inverse trend existed for an increasing number of LEs and a decreased score for SF-12 PCS, SF-12 MCS and WEMWEBS (Table 4). Kruskal–Wallis H analysis confirmed that the cumulative occurrence of LEs was associated with significantly different mental health (SF-12 MCS) (*p* < 0.01) and WEMWBS scores (*p* < 0.01), but not with significantly different physical health (SF-12 PCS) scores. Subsequent, post hoc analysis with Bonferroni correction showed that individuals who experienced 3 or 4 LEs had significantly lower SF-12 MCS scores than those who had no LEs (*p* < 0.01); and significantly lower WEMWBS than those who had no LEs (*p* < 0.01) or 1 LE (*p* < 0.05) (Table 4).

**Table 4:** Physical and mental health and well-being scores by cumulative number of LE/s

Number of life events^a^	Total frequency (n)	Health outcome measures^a^		
		SF-12 MCS	SF-12 PCS	WEMWBS
		Mean (SD)		
0	214	52.8 (9.1)	43.8 (14.2)	52.2 (8.1)
1	338	50.4 (11.0)	43.3 (13.8)	50.7 (9.2)
2	260	49.3 (11.9)	41.1 (15.3)	49.7 (10.3)
3	159	47.2 (12.8)*	41.3 (15.3)	46.7 (11.8)**^,†^
4	76	44.7 (14.3)*	38.8 (15.0)	45.9 (13.0)**^,†^
5	18	49.3 (11.4)	41.0 (15.9)	46.4 (14.8)
6	4	47.3 (8.9)	51.3 (9.1)	53.0 (7.6)
7	1	52.0	54.5	57.0

* Significantly lower SF-12 MCS than 0 LE (*p* = 0.001)

** Significantly lower WEMWBS than 0 LE (*p* ≤ 0.001)

^†^Significantly lower WEMWBS than the occurrence of 1 LE (*p* < 0.05)

^a^Data collected at wave 3, 2011

### Analysis of variance

The proportion of variance, which could be attributed to, the first stages of the multivariate models (demographic variables) were of the lowest proportion (SF-12 MCS = 0.030; SF-12 PCS = 0.154; and WEMWBS = 0.020). Within the second stage of the models, which included baseline health measures, results showed that the proportion of variance increased by the widest margin for each health outcome measure (SF-12 MCS = 0.111; SF-12 PCS = 0.360; and WEMWBS = 0.137). For the final stage of the model, LEs were included, with results showing that the proportion of variance was the greatest for each model (SF-12 MCS = 0.194; SF-12 PCS = 0.418; and WEMWBS = 0.217).

### General linear models

#### SF-12 MCS

Results from the first GLM showed that for the SF-12 MCS, experience of five LEs had a statistically significant (*p* < 0.05) negative impact on mental health. In order of mean difference: a relationship breakdown (−7.3, *p* < 0.01); a health event (−6.0, *p* < 0.01); being the victim of a crime (−5.4, *p* < 0.01); bereavement (−2.3, *p* < 0.05); and house move (−2.1, *p* < 0.05) (Table 5). Two LE had a significantly positive impact on SF-12 MCS: a new job/promotion resulted in a +4.9 mean difference between groups (*p* < 0.01) and parenthood resulted in a +3.6 (*p* < 0.05) difference (Table 5).

**Table 5:** General linear model for each of the three dependent health variables and occurrence of each LE

Life events^b^	Health outcome measures^a^											
	SF-12 MCS				SF-12 PCS				WEMWBS			
	Yes	No	Between group differences	*p* value	Yes	No	Between group differences	*p* value	Yes	No	Between group differences	*p* value
Health event	45.01 (43.8 to 46.3)	51.03 (50.3 to 51.8)	−6.0 (−7.5 to −4.5)	0.000*	36.09 (34.8 to 37.4)	44.41 (43.6 to 45.2)	−8.3 (−9.9 to −6.8)	0.000*	45.62 (44.5 to 46.7)	50.68 (50.0 to 51.3)	−5.1 (−6.4 to −3.7)	0.000*
New job	53.96 (51.4 to 56.5)	49.07 (48.4 to 49.7)	4.9 (2.3 to 7.5)	0.000*	44.64 (41.9 to 47.3)	41.99 (41.3 to 42.9)	2.7 (−0.2 to 5.5)	0.065	53.34 (51.1 to 55.6)	49.03 (48.4 to 49.6)	4.3 (2.0 to 6.6)	0.000*
Unemployment	51.65 (49.3 to 54.0)	49.25 (48.6 to 49.9)	2.4 (−0.9 to 4.9)	0.059	42.93 (40.4 to 45.5)	42.11 (41.4 to 42.8)	0.8 (−1.8 to 3.4)	0.545	50.06 (48.0 to 52.2)	49.3 (48.7 to 49.9)	0.8 (−1.4 to 3.0)	0.480
Parenthood	52.83 (50.0 to 55.7)	49.23 (48.6 to 49.9)	3.6 (0.7 to 6.5)	0.016*	44.85 (41.8 to 47.9)	42.05 (41.3 to 42.8)	2.8 (−0.3 to 5.9)	0.080	51.21 (48.6 to 53.8)	49.26 (48.7 to 49.8)	2.0 (−0.7 to 4.6)	0.147
Relationship break down	42.41 (39.3 to 45.5)	49.75 (49.1 to 50.4)	−7.3 (−10.6 to −4.1)	0.000*	40.56 (37.2 to 43.9)	42.29 (41.6 to 43.0)	−1.7 (−5.2 to 1.7)	0.328	44.80 (42.1 to 47.6)	49.56 (49.0 to 50.1)	−4.8 (−7.6 to −1.9)	0.001*
Bereavement	47.75 (46.5 to 49.0)	50.03 (49.3 to 50.8)	−2.3 (−3.8 to −0.8)	0.002*	40.61 (39.3 to 42.0)	42.75 (41.9 to 43.5)	−2.1 (−3.7 to −0.6)	0.008*	48.16 (47.0 to 49.33)	49.8 (49.1 to 50.4)	−1.6 (−2.9 to −0.3)	0.017*
Marriage	52.46 (46.8 to 58.1)	49.40 (48.7 to 50.0)	3.1 (−2.6 to 8.7)	0.290	44.87 (38.9 to 50.9)	42.15 (41.5 to 42.8)	2.7 (−3.3 to 8.8)	0.377	54.93 (50.0 to 59.9)	49.30 (48.7 to 49.9)	5.6 (0.6 to 10.6)	0.027*
Victimisation	44.45 (42.0 to 46.9)	49.81 (49.1 to 50.5)	−5.4 (−7.9 to −2.9)	0.000*	41.83 (39.2 to 44.4)	42.2 (41.5 to 42.9)	−0.4 (−3.0 to 2.3)	0.780	45.04 (42.9 to 47.2)	49.7 (49.1 to 50.3)	−4.6 (−6.9 to −2.4)	0.000*
Behavioural problem with child	49.64 (45.7 to 53.6)	49.46 (48.8 to 50.1)	0.2 (−3.8 to 4.2)	0.931	41.12 (36.9 to 45.4)	42.12 (41.4 to 42.8)	1.0 (−5.3 to 3.3)	0.651	47.34 (43.8 to 50.8)	49.4 (48.8 to 49.9)	−2.0 (−5.6 to 1.5)	0.264
Housing improvement	49.29 (48.3 to 50.2)	50.26 (49.3 to 51.2)	1.0 (−2.3 to 0.4)	0.150	41.45 (40.4 to 42.5)	43.08 (42.1 to 44.1)	−1.6 (−3.1 to −0.2)	0.025*	48.52 (47.7 to 49.3)	50.81 (50.0 to 51.6)	−2.3 (−3.4 to −1.1)	0.000*
House move	48.07 (47.0 to 49.1)	50.15 (49.4 to 50.9)	−2.1 (−3.4 to −0.8)	0.002*	42.21 (41.1 to 43.3)	42.08 (41.2 to 42.9)	0.1 (−1.3 to 1.6)	0.866	47.55 (46.6 to 48.5)	50.22 (49.5 to 50.9)	−2.7 (−3.8 to −1.5)	0.000*

^a,b^Data collected at Wave 3, 2011; demographic characteristics collected at follow-up Wave 3 survey (gender, age and highest educational attainment) and baseline measures of health (WEMWBS, SF-12 PCS, SF-12 MCS) collected at Wave 2 survey were included in the models as controls

#### SF-12 PCS

For SF-12 PCS three LEs were associated with a statistically significant negative mean difference between groups: the occurrence of a health event (−8.3, *p* < 0.01), bereavement (−2.1, *p* < 0.01) and housing improvements (−1.6 *p* < 0.05) (Table 5). This would suggest that there are fewer direct links between life events and physical health than there are with mental health. The remaining LEs did not show statistical significance between groups although three LEs (new job, parenthood and marriage) showed impacts in a positive direction and one (relationship breakdown) in a negative direction. Very little difference was observed in SF-12 PCS scores between groups for the experience of the occurrence of a house move, unemployment, victimisation and behavioural problems with a child (Table 5).

#### WEMWBS

Looking at WEMWBS scores, six of the LEs were found to have a significant association with poorer mental well-being. In order of scale of impact, these were: experience of a health event (mean difference between groups −5.1, *p* < 0.01); relationship breakdown (−4.8, *p* < 0.01); victimisation (−4.6, *p* < 0.01); a house move (−2.7, *p* < 0.01); housing improvement (−2.5, *p* < 0.01); and bereavement (−1.6, *p* < 0.05) (Table 5). Two LEs were found to have a significant positive effect on WEMWBS; marriage [significant mean difference between groups of +5.6 (*p* < 0.05)] and experience of a new job/promotion, [mean difference between groups of +4.3 (*p* < 0.01)] (Table 5).

## Discussion

To our knowledge this is one of the few studies to investigate the impact of a range of individually and cumulatively occurring LEs on mental and physical health and mental well-being. Our findings confirm that health outcomes deteriorate in line with accumulating LEs, but that the most significant drop occurs where people experience three or four LEs over a 3 year period.

Results showed the most commonly occurring LEs were a serious health event, bereavement, house improvement or house move. With 80 % of the sample experiencing one or more LEs during the study period, LEs are shown to be highly prevalent among the adults in this deprived population. We found that only particular LEs have significantly detrimental impacts on physical health (health event, bereavement and housing improvements), mental health (bereavement, victimisation, health event, relationship breakdown and house move) and mental well-being (health event, victimisation, housing improvement, house move, bereavement and relationship breakdown). In the case of mental health, the negative effects of LEs were greater in magnitude than the positive effects of others. However, for mental well-being, the positive and negative effects of different life events were more similar in magnitude.

Two LEs were associated with poorer outcomes across all three health measures: a serious health event (affecting the respondent or another household member) and bereavement (death of someone close). This highlights the particular importance of having adequate support for those experiencing or caring for others with health problems and coping with bereavement [30].

Findings also showed that both housing improvements and a house move had a significantly negative impact on mental well-being scores, perhaps contrary to many expectations. Other studies have found little association between housing improvements and health [31–33]. As with the other LEs it is plausible to assume that housing events are complex and their occurrence represents a challenging time. Therefore, the potentially beneficial health-related outcomes expected as a result of housing improvements, redevelopment and rehousing may only transpire as a result of longer term follow-up. The current study has not allowed for ‘time since event’ to be incorporated into our analysis to see whether the negative impacts observed here occur close to the LEs themselves.

One LE was found to have a significantly positive impact on mental health and well-being scores—‘getting a new job/promotion’—reinforcing previous findings about the importance of employment to good health and well-being [11]. However, relatively few respondents experienced this LE, despite low levels of employment at baseline. Marriage has little or no effect on mental and physical outcomes although it did have a significantly positive impact on mental well-being. Similarly, parenthood did not have a significant impact on physical health or mental well-being but it did have a significantly positive impact on mental health. For ‘undesirable’ LEs, as previously stated we can confirm that health events do have negative impacts across all three outcomes, and that victimisation has a significantly negative effect upon both mental well-being and mental health. We did not find evidence for negative effects from unemployment or child behavioural problems.

### Strengths and limitations

A strength of this study is the longitudinal dataset, enabling us to control for baseline health scores. As LEs are complex and multi-faceted, but due to the nature of the questions asked within this study a limitation was not being able to unpick what element of the LE was responsible for the impact on health. Secondly, it was not possible to unravel the mechanisms and pathways through which experiencing particular LEs might impact on health directly or indirectly (e.g. through the uptake of unhealthy behaviours as coping strategies). Thirdly, our findings may be affected by negative response bias in that those with existing poor health may over-report negative aspects of their lives. However, the importance of this may be limited [34].

## Conclusions

The findings from the current study highlight both the detrimental and beneficial impacts that particular LEs can have on the health and wellbeing of individuals. Not all LEs have effects as expected, and in particular, what appear to be positive housing-related events were found to have negative effects on health and well-being. Further research is required to disentangle the complexity of the occurrence of LEs and the ways in which they affect the health and well-being of the individual.

## Abbreviations

AE: Anne Ellaway
AK: Ade Kearns
CC: Claire Cleland
CT: Carol Tannahill
LE/s: life event/s
MCS: mental component score
PCS: physical component score
SD: standard deviation
SLC: Scottish leaving certificate
WEMWBS: Warwick–Edinburgh mental well-being scale
